# Comparison of ECG Saline-Conduction Technique and ECG Wire-Based Technique for Peripherally Inserted Central Catheter Insertion: A Randomized Controlled Trial

**DOI:** 10.3390/s24030894

**Published:** 2024-01-30

**Authors:** Giuseppe Gullo, Pierre Frossard, Anaïs Colin, Salah Dine Qanadli

**Affiliations:** 1 Lausanne University Hospital, Department of Diagnostic and Interventional Radiology, Rue du Bugnon 46, 1011 Lausanne, Switzerland; pierre.frossard@chuv.ch (P.F.); anais.colin@chuv.ch (A.C.); 2 Faculty of Biology and Medicine, University of Lausanne, 1015 Lausanne, Switzerland; 3 Clinical Research Unit, Riviera-Chablais Hospital, 1847 Rennaz, Switzerland

**Keywords:** central venous catheters, electrocardiography, hemostasis, interventional radiology, superior vena cava, catheterization, peripheral

## Abstract

(1) Background: The peripherally inserted central catheter (PICC) is commonly used in medicine. The tip position was shown to be a major determinant in PICC function and related complications. Recent advances in ECG guidance might facilitate daily practice. This study aimed to compare two ECG techniques, in terms of their tip-position accuracy, puncture site layout, and signal quality; (2) Methods: This randomized open study (1:1) included 320 participants. One PICC guidance technique used ECG signal transmission with saline (ST); the other technique used a guidewire (WT). Techniques were compared by the distance between the catheter tip and the cavoatrial junction (DCAJ) on chest X-rays, insertion-point hemostasis time, and the extracorporeal catheter length between the hub and the insertion point; (3) Results: The mean DCAJs were significantly different between ST (1.36 cm, 95% CI: 1.22–1.37) and WT (1.12 cm, 95% CI: 0.98–1.25; *p* = 0.013) groups. When DCAJs were classified as optimal, suboptimal, or inadequate, the difference between techniques had limited clinical impact (*p* = 0.085). However, the hemostasis time at the puncture site was significantly better with WT (no delay in 82% of patients) compared to ST (no delay in 50% of patients; *p* < 0.001). Conversely, ST achieved optimal and suboptimal extracorporeal lengths significantly more frequently than WT (100% vs. 66%; *p* < 0.001); (4) Conclusions: ECG guidance technologies achieved significantly different tip placements, but the difference had minimal clinical impact. Nevertheless, each technique displayed an important drawback at the PICC insertion point: the extracorporeal catheter was significantly longer with WT and the hemostasis delay was significantly longer with ST.

## 1. Introduction

The first peripherally inserted central catheter (PICC) was described in 1912 by Bleichröder [1]. Implementations of the device have been marked by several innovations, and ECG guidance is one of the more longstanding.

The first articles to mention ECG guidance were published in 1948–1949. The technique was presented with both saline-signal and wire-signal conduction methods [2,3]. From 1950 to 2000, at least 30 studies were published on the subject [4], but regular use of the PICC technique did not gain traction until 2008 [5]. As a reminder, radiology departments became overwhelmed because they faced an exponential increase in PICC procedures (>20% annually from 1995 to 2003) [6], resulting in delayed patient care and reduced discharge rates for outpatient therapies.

The principal strengths of ECG is that it improves PICC placement by eliminating X-rays, thus, allowing confirmation of correct placement. ECG guidance has been recognized as a valid alternative since 2015 [7] and currently 62% of non-fluoroscopy specialists use it [8].

The position of the PICC relative to the cavoatrial junction (CAJ) is a major predisposing factor for complications [9]. When clinicians can ensure that catheters terminate in the vena cava rather than a midclavicular vein, the rates of symptomatic PICC-related deep vein thrombosis are reduced by 40% [10]. Conversely, a tip positioned after the CAJ may cause cardiac arrest or arrhythmias, due to mechanical irritation of the endocardium [11,12].

Different studies compared ECG guidance to blind techniques (BT), in a meta-analysis [13], the odds ratio for better positioning accuracy was 2.88 with ECG guidance versus BT. However, we lack studies that compare different ECG-guidance technologies.

Therefore, this prospective randomized controlled study aimed to compare the ECG saline conduction technique (ST) and the ECG wire-based technique (WT) for PICC insertion, based on the precision of the tip position, the puncture site layout, and the signal quality.

## 2. Materials and Methods

### 2.1. Patients

The Swiss Association of Research Ethics Committees approved the study protocol (BASEC 2020-00583) and it was registered on ClinicalTrials.gov (NCT04466332).

Written informed consent was obtained from each patient. All consecutive patients over 18 years old that were referred to the radiology department for PICC insertion were recruited for study enrollment.

Candidates were excluded when they had impaired heart rhythms that changed the P-wave presentation (e.g., atrial fibrillation or flutter, severe tachycardia, pacemaker) or weighed over 150 kg (maximal table load)

### 2.2. PICC Placement

Insertions were performed by a PICC-team of two radiologists and five technicians. All investigators had at least four years of experience in PICC placement. Operators were instructed to follow standardized ECG-guidance procedures. All operators had previously performed over 100 PICC insertions under ECG (50 with ST and 50 with WT).

All PICC insertions were assessed with the same interventional radiology instrument (Artis-Zee, Siemens Healthcare GmbH, Munich, Germany). Chest X-rays (CXR) were used for comparisons. All patients were placed in a supine position, and CXRs were acquired in a postero-anterior view with deep inspiration. Venous access was achieved with the linear probe part of the Site~Rite8^®^ ultrasound system (Becton Dickinson, Franklin Lakes, NJ, USA). The insertion procedure was performed with the maximal sterile barrier approach. Before insertion, external ECG electrodes were placed on the chest. The insertion procedure was previously described [14,15].

### 2.3. Wire-Based Technique (WT)

Prior to insertion, a 4F PowerPICC-SOLO-2™ catheter (Becton Dickinson, Franklin Lakes, NJ, USA) was trimmed to the anticipated required length, based on anthropometric parameters. The length was approximated as: the distance between the insertion site and the axillary crease + the distance between the axillary crease and the sternal notch + 13 cm [16].

**Figure 1:**
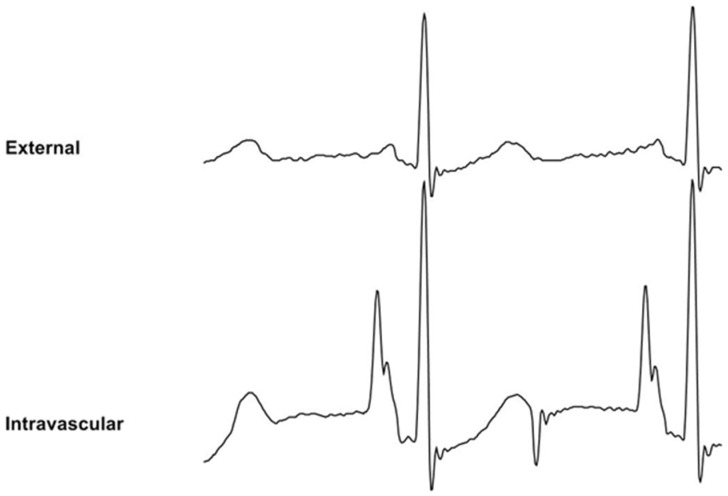
Wire-based technique (WT) displaying external ECG (upper trace) and intravascular ECG (lower trace). The intravascular ECG shows an increased P-wave relative to the external ECG.

Definitive PICC positioning was performed with ECG technology (Site~Rite8^®^ integrated Sherlock-3CG™ Diamond-Tip Confirmation System; Becton Dickinson, Franklin Lakes, NJ, USA), which included two successive phases. The navigation phase consisted of advancing the PICC from the insertion site to the central circulation. For magnetic navigation, a Y-shield was installed on the chest to serve as a low-field magnetic complex. The position of the catheter tip relative to the shield was tracked electronically and erroneous locations (e.g., jugular vein or contralateral crossover) could be identified. A monitor displayed a trace for real-time tracking, until the tip moved toward the superior vena cava (SVC). At the SVC level, the second phase consisted of positioning the catheter at an adequate central location. Conductivity was ensured through the wire, and a monitor displayed both surface and intracavitary real time ECG traces to allow the practitioner to adjust the PICC tip at the cavoatrial junction (CAJ). When the catheter reached the CAJ, the P-wave showed its maximal amplitude without initial negative deflection (Figure 1). After implanting the tip at the CAJ, the puncture site was dressed with a catheter stabilization device.

### 2.4. Saline Conduction Technique (ST)

**Figure 2:**
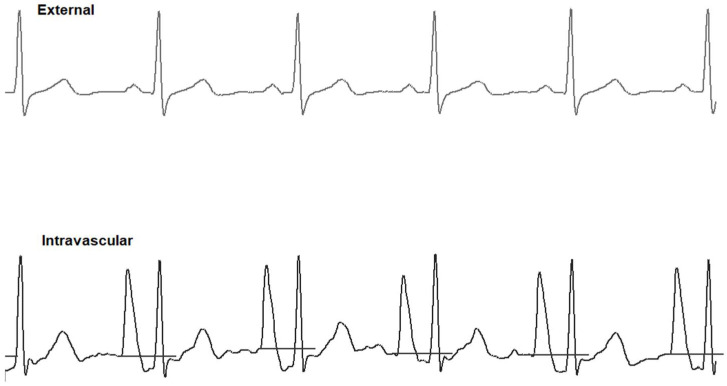
Saline conduction technique (ST) displaying external ECG (upper trace) and intravascular ECG (lower trace). The intravascular ECG shows an increased P-wave relative to the external ECG.

The saline conduction technique applied the same venous access and site dressing as those described for the WT technique. However, the 4F Lifecath CT-PICC Easy^®^ catheter (Vygon, Ecouen, France) was not trimmed before advancing it into the central circulation. Instead, the catheter position was approximated by identifying the point where a P-wave modification should occur (Pilot Tip-Location-System; Vygon, Ecouen, France). Tip navigation relied on the shape interpretation of the QRS signal, which allowed the operator to differentiate between central and non-central veins. Centrally, positioning at the CAJ was performed the same way as described for the WT technique, and conductivity was ensured with 0.9% saline (Figure 2). The distal (extracorporeal) part of the PICC was cut without withdrawing it, then the removable part of the catheter was connected.

### 2.5. Data Collection and Statistical Analysis

Open-label randomization was performed using a 1:1 allocation ratio with a computer random number generator and allocation concealment through sequentially numbered opaque sealed envelopes. A person not involved with patient recruiting prepared this.

Technical success was defined as the placement of the PICC through the selected access vein into the central venous circulation. The CXRs for successful procedures were anonymized, and tip positions were independently assessed by the members of the PICC-Team, who were blinded to the guidance technique. Interpretation was standardized by requiring the assessors to complete joint training with a set of patient images (not included in the study), conducted on a Clear-Canvas Workstation (Clear-Canvas Inc. Synaptive Medical Inc., Toronto, ON, Canada). Disagreements were resolved by consensus in a supplementary joint reading session.

**Figure 3:**
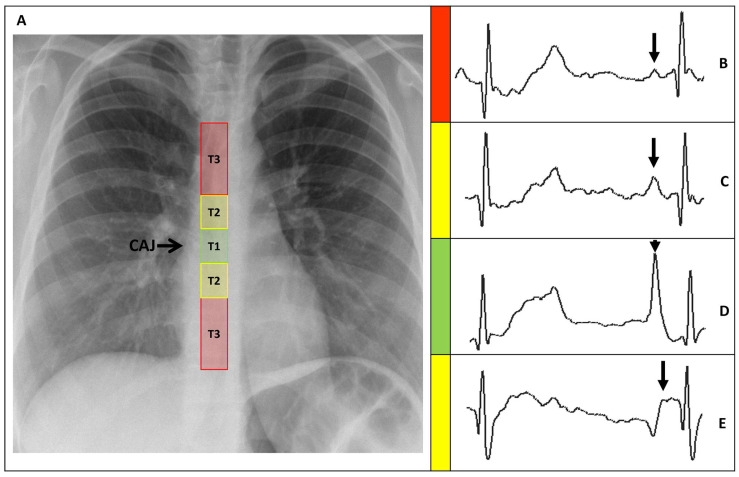
(**A**) Chest X-ray showing the CAJ localization. The tip of the catheter in T1 (within 1 cm of the CAJ) is considered optimal (green zone). The tip in T2 (within 1–3 cm of the CAJ) is considered suboptimal (yellow zone). The tip in T3 (more than 3 cm below the CAJ or not in the SVC) is considered inadequate and needs to be repositioned (red zone). (**B**) Intracavitary electrocardiogram. A catheter tip in T3, distant from the SVC, will feature a trace similar to the superficial ECG trace, with a P-wave lower than the Twave immediately preceding it. (**C**) Intracavitary electrocardiogram. A catheter tip in T2, nearing the CAJ, will feature a trace with increasing P-wave, equaling the Twave immediately preceding it. (**D**) Intracavitary electrocardiogram. A catheter tip in T1, positioned at the CAJ level, will feature a P-wave with maximal amplitude without initial negative deflection. (**E**) Intracavitary electrocardiogram. A catheter tip in T2-T3, beyond the CAJ, will present a decreasing P-wave and negative deflection. CAJ, cavoatrial junction (open arrow); SVC, superior vena cava; P-wave (closed arrow).

PICCs positions were classified, based on the distance between the catheter tip and the CAJ (DCAJ), as previously described and imaged [14]. The classifications were: T1: a final tip position of ±1 cm from the CAJ (optimal); T2: a tip position 1–3 cm above or below the CAJ (suboptimal); and T3: a tip position >3 cm below the CAJ or not in the SVC (inadequate, required repositioning) (Figure 3).

**Figure 4:**
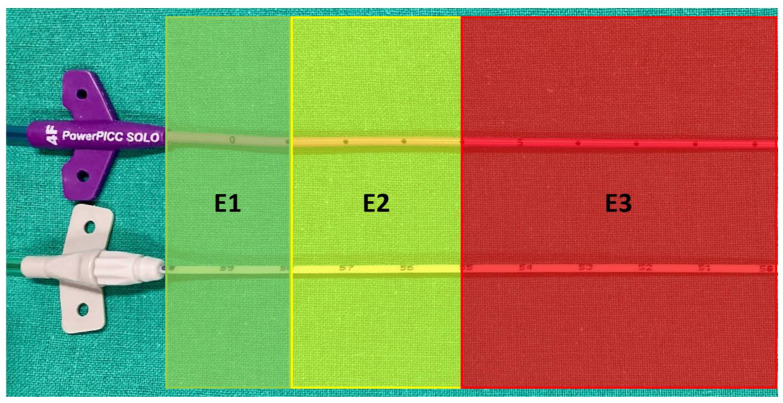
PICC hub side at insertion point. Outside catheter length classified as: E1 from 0 to 2 cm apart from the hub (optimal); E2 from 2 to 5 cm apart from the hub (suboptimal); and E3 > 5 cm apart from the hub (inadequate, requiring recut). Upper catheter: Wire-based technique (WT); lower catheter: Saline conduction technique (ST).

The PICC puncture sites were also classified based on the extracorporeal catheter length, then measured between the catheter hub and the insertion point. The classifications were: E1: 0–2 cm, which equaled ±1 cm around the “0” position recommended in the BARD instructions (optimal) [17]; E2: 2–5 cm (suboptimal); and E3 > 5 cm (inadequate, required a recut) (Figure 4).

The pull-out distance from the central position was defined as the intravascular distance between the maximal positive P-wave and the initial negative P-wave deflection. It was measured using the extracorporeal catheter graduation during the procedure.

**Figure 5:**
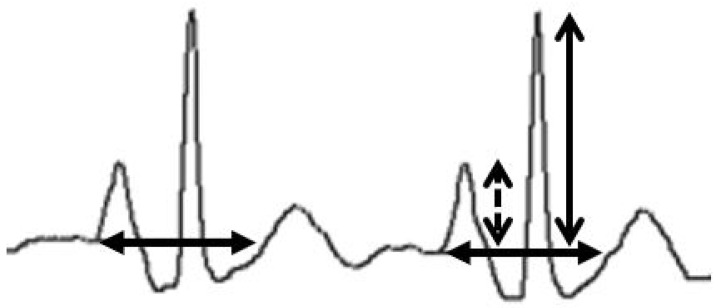
Wave ratio calculation. The maximal P-wave (dashed arrow) is divided by the maximal QRS height (open arrow), both arising from the isoelectric line (closed arrow; considered as the prolongation of the TP interval immediately preceding the P-wave as displayed on the Pilot Tip-Location-System).

A wave ratio (P/QRS) was calculated. It represents the criterion for determining a significant change in P-wave which indicates that the PICC tip is near the CAJ. It is obtained by dividing the maximal P-wave by the maximal QRS height, both arising from the isoelectric line [18]. (Figure 5).

Hemostasis was dichotomized according to whether compression at the insertion site was necessary to achieve hemostasis. Direct hemostasis was achieved without compression; delayed hemostasis was after one or more minutes of compression.

Based on experience, and as described in previous studies [15,16], the estimated success rate with the WT technique was 85%. The ST success rate was expected to be within 10% of the WT success rate (95%). Therefore, the estimated sample size for the Pearson’s chi-squared test (80% power and 5% alpha) was N = 320, with two groups of 160 participants.

Statistical analyses were performed with STATA v.16.1 (Stata Corp., College Station, TX, USA). Fisher’s exact or chi-squared tests were performed for categorical variables. The Mann–Whitney U test was performed for continuous variables. Data distributions were verified with the Shapiro–Wilk W test.

## 3. Results

**Figure 6:**
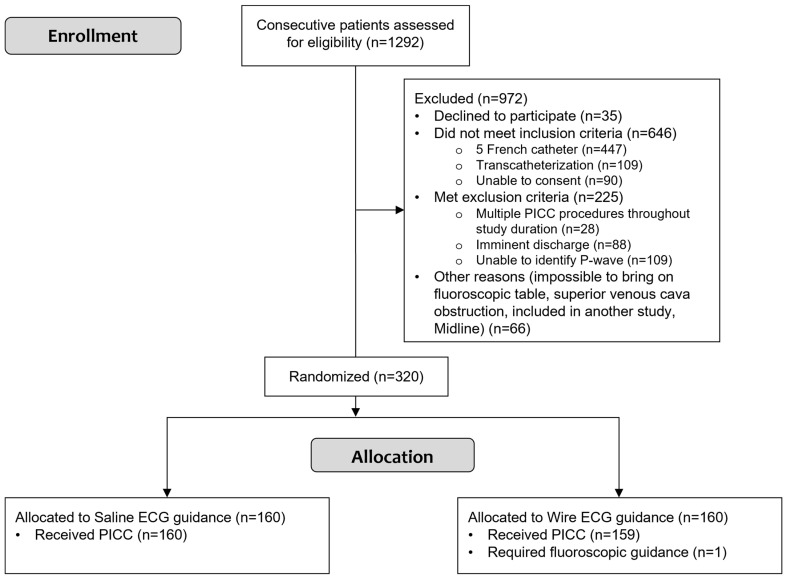
Study flowchart shows participant selection and allocation. PICC: peripherally inserted central catheter.

From September 2020 to August 2021, 1292 patients were screened. Of these, 160 were assigned to each group (Figure 6). The study was completed regularly according to plan with no serious adverse events.

PICCs were successfully placed in 319 patients (194 men and 125 women; mean age 61.5 years). In the remaining patients, the PICC procedure was converted to guided fluoroscopic insertion, due to an unknown central-venous obstruction that precluded PICC progression. This patient was excluded from the analysis.

The baseline demographics were not significantly different between the groups (Table 1). PICC placement indications included: antibiotic therapy (72.1%), repeated blood draws (6.9%), chemotherapy (10.3%), and parenteral nutrition (6.3%). Access was achieved through the basilic vein (69.6%) or the brachial vein (30.4%).

**Figure 7:**
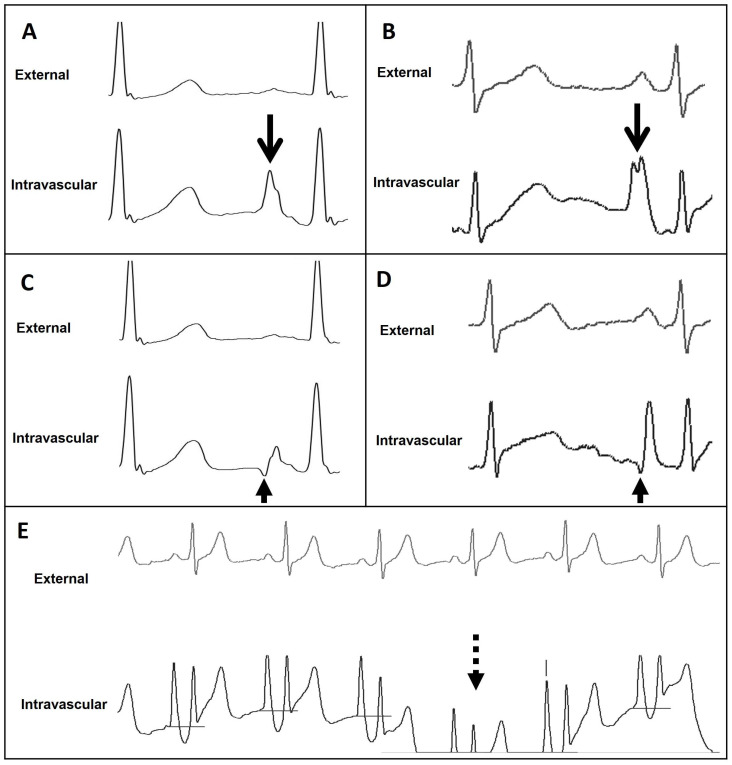
External and intravascular ECG trace of the Wire-based technique (WT) and Saline conduction technique (ST). (**A**) WT trace featuring maximal P-wave at the level of CAJ (open arrow). (**B**) ST trace featuring maximal P-wave at the level of CAJ (open arrow). (**C**) WT trace featuring initial negative deflection just beyond the level of CAJ (closed arrow). (**D**) ST trace featuring initial negative deflection just beyond the level of CAJ (closed arrow). (**E**) ST trace displaying important drifts (dashed arrow). CAJ, cavoatrial junction.

PICCs were successfully placed (i.e., technical success) in 100% of the ST group and 99.4% of the WT group (Table 2 and Figure 7). The overall mean procedural time was 38.2 min. The global mean wave ratios were above 80% in both groups (ST: 87.7% and WT: 81.5%).

During the navigation phase, a J-wire was required for central catheter positioning [19] 16 times (12 for ST and 4 for WT), representing 5% of all procedures (Table 2).

During the procedure (i.e., before the final CRX), PICCs that were too long had to be readjusted (E3). Intraprocedural recuts required a whole-catheter pull-out, a recut, and a reinsertion in a new navigation phase. No E3s occurred with ST PICCs. E3s occurred in 80 cases with WT PICCs (55.2%), including 48 right-side PICCs and 32 left side PICCs (48.5 vs. 69.6%; Table 2).

The final tip-position classifications were similar between the groups (*p* = 0.086). After positioning, PICCs were classified as T3 (i.e., inadequate placement that required repositioning) in 13 ST cases (8.1%) and 14 WT cases (8.8%). Among these, 3 ST and 2 WT PICCs were looped, and 4 WT PICCs were short; thus, 3 ST and 6 WT catheters were inserted at least 3 cm from the CAJ. The remaining class-T3 PICCs (10 ST and 8 WT) were too long (Table 3).

The overall success rates for T1 and T2 placements were similar between the two techniques (91.9% vs. 91.2%). The ST approach achieved optimal tip positions (T1) in 58 cases (36.3%) and suboptimal positions (T2) in 89 cases (55.6%). The WT approach achieved 76 T1 positions (47.8%) and 69 T2 positions (43.4%; Table 3).

**Figure 8:**
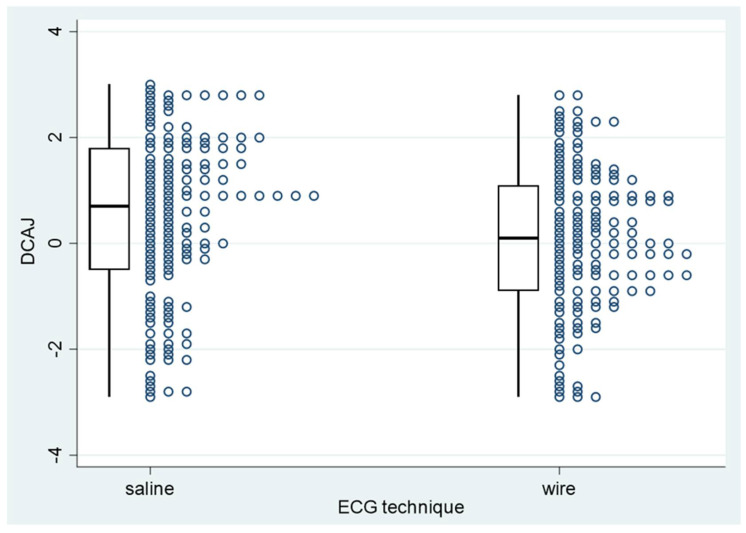
PICC−to−cavoatrial junction distance for PICC placements with saline or wire guidance. PICC: peripherally inserted central catheter; DCAJ: distance between the catheter tip and the cavoatrial junction.

Among PICCs classified as T1 or T2, the absolute mean DCAJ distances were 1.36 cm in the ST group (95% CI: 1.22–1.50 cm) and 1.12 cm in the WT group (95% CI: 0.98–1.25 cm; *p* = 0.0012; Table 3 and Figure 8). Among T1 and T2 PICCs, those placed with ST were longer than those placed with WT (Figure 4). For T1 and T2 PICCs, the mean pull-out length was 1.66 cm (95% CI: 1.56–1.77 cm; range: 0.5–5 cm; Table 3).

At the insertion point (Table 4), the extracorporeal catheter lengths were classified at first insertion. The WT approach achieved 40 (27.6%) E1, 56 (38.6%) E2, and 49 (33.8%) E3 insertions. WT catheters had a mean extracorporeal length of 4.36 cm (95% CI: 3.96–4.76 cm; range: 0–11 cm). After the intraprocedural recuts, no catheters were classified as E3. In the ST group, all 147 catheters in T1 and T2 positions were classified as E1. In the WT group, 116 catheters in T1 and T2 positions (80%) were classified as E1 and 29 (20%) were classified as E2. The final mean extracorporeal catheter lengths were 1.61 cm (95% CI: 1.44–1.77 cm; range 0–4 cm) for the WT group and 0.01 cm (95% CI: −0.005–0.03 cm; range 0–4 cm) for the ST group (*p* < 0.001).

Direct hemostasis was achieved in 120 (82.8%) WT insertions, but only in 50.3% of ST insertions (*p* < 0.001; Table 4).

## 4. Discussion

Several prior studies have compared ECG-based techniques for central venous catheter insertions. However, either they compared devices other than PICCs or they were quasi-experimental. Our results showed that both techniques had high technical success rates; consistent with the 91.7% demonstrated in a previous study [20]. There was no significant difference in T1/T2/T3 categories between groups. Globally, we found optimal and suboptimal positioning in >90% of cases. Malpositions that required corrections occurred in around 8% of the groups. Previously reported misplacement rates were around 4% [21]. Positioning errors due to the loss of catheter rigidity (i.e., guidewire removal) mentioned in precedent studies [22,23] were not evident in our study. WT PICCs tended to be shorter than ST PICCs, but the distributions of T1-T2-T3 categories were similar between groups.

### 4.1. Navigation Capacities

The studied techniques had different navigation capacities. ST required a larger number of supplemental J-wire interventions than WT (12 vs. 4) [19], but the difference was not significant. Magnetic and ECG navigation have never been studied specifically; our findings of no significant differences between groups in J-wire interventions or in the T1-T2-T3 categories suggested that neither magnetic navigation nor ECG navigation showed superiority.

The WT required significantly more intraprocedural catheter recuts (55%) than the ST (0%). This difference was clearly linked to the time that the catheter was trimmed (pre- vs. post-insertion). Intraprocedural recuts lead to multiple insertions, and the infection risk is questionable. The few studies that addressed this point concluded that readjustments were not significantly correlated to an increased risk of central line-associated bloodstream infection (CLABSI) or thrombosis [24]. We observed that, in the WT subgroup, catheter recuts were necessary significantly more frequently on the right side than on the left side. Considering that the catheter must be cut prior to the WT procedure, this finding emphasized the critical need to differentiate between right and left sides in estimating catheter lengths and the proper limits of surface anatomy.

### 4.2. Positioning Capacities

The T1 and T2 optimal and suboptimal central tip positions were significantly different between the ST and WT groups (mean DCAJ: 1.36 and 1.12 cm, respectively). Considering the distribution of the catheter DCAJ, ST-placed PICCs were longer than WT-placed. This difference might be explained by differences in procedure. For hub fixation, ST required approximatively three more centimeters than WT, due to the minimal length needed to cut the catheter, connect the hub, and readvance the entire catheter into its final position.

The ST procedure duration was slightly longer than the WT, but the global time was comparable to those reported previously [25]. The difference we observed might be explained by a qualitative difference in signal stability. As in two other studies [26,27], we observed drifts due to P-wave interference with the ST. Moreover, the WT navigation signal was easier to interpret because a monitor displayed its path. In contrast, the ST signal had to be interpreted to identify a modification in the QRS complex, which was less intuitive [28].

The ST and WT technologies had comparable ECG signals amplitudes. The P/QRS were equivalent in optimal and suboptimal positions, with a mean of around 80%, similar to the 87% optimal value reported previously [29]. Conversely, two previous studies [27,30] noticed a more pronounced signal amplitude when comparing WT to ST, WT showed better quality. Cheng et al. found that ST showed a better-quality pattern than WT, but they used NaHCO3, which has better conductivity properties than saline [26].

Independent of the ECG technique, a P/QRS ratio >40% (5th percentile) ensured a centrally positioned tip within 3 cm of the CAJ. Our technical success rates were equivalent to the 99% success rate obtained by Dong et al. [18], with a P/R ratio >50%.

The mean pull-out length was 1.6 cm, with no difference between groups. This length was comparable to the 2 cm already mentioned [31].

### 4.3. Hemostasis and Extracorporeal Catheter at Insertion Point

At the first PICC insertion, the mean extracorporeal catheter lengths were 4.36 cm for WT and 0.01 cm for ST. All ST insertions were classified as E1. However, only 40 (27.6%) WT insertions were classified as E1; 56 (38.6%) were classified as E2, and 49 (33.8%) were classified as E3 (6 catheters were recut, due to operator choice, and they were classified as E2). After recuts, no WT insertions were classified as E3, and 80% were classified as E1. The mean WT extracorporeal catheter length was reduced to 1.61 cm.

Few authors have considered how long the extracorporeal catheter should be. It remains controversial whether phlebitis is associated with catheter movements and whether the length facilitates nursing care [32]. Baxi et al. [24] showed that a single post-procedural adjustment was protective against CLABSI. Thus, it may be necessary to ensure a reasonable extracorporeal catheter length. Elli et al. [33] recommended that the extracorporeal length should be 1 cm to facilitate nursing care, such as dressings, disinfection, and stabilization.

It is also important to consider hemostasis. Only Itkin et al. addressed this issue, and they concluded that post-procedure bleeding rates were not significantly different between straight and reverse-tapered devices [34]. Although bleeding was not thoroughly described, our results differed from theirs. We concluded that the reverse-tapered device (PowerPICC-SOLO-2™) largely contributed to hemostasis, compared to the straight device (Lifecath-CT-PICC-Easy^®^). To achieve better hemostasis with a straight device, cyanoacrylate could be used, as it combines enhanced hemostasis capacities with infection prevention and stabilization [35].

### 4.4. Accessibility and Usability

The WT is restricted to specific PICC devices marketed by Becton Dickinson. It is also limited to the adult population (the Y shield is not adapted for infants) [36]. The branded tip confirmation system is simple and intuitive, it is also a full standalone system with ultrasound capacities. The buttons on the ultrasound probe allow navigating through the different features shown on the tip confirmation system screen. This feature is highly functional as it enables a single clinician to operate autonomously while ensuring a sterile working environment.

The ST is significantly more accessible due to its compatibility with all marketed devices, without any brand limitations, for both infants and adults [36]. However, it lacks in usability as the operator must be assisted to interact with the Pilot Tip-Location-System. Moreover, the displayed trace is more sensitive to drifts.

### 4.5. Limitations

This study had several limitations. First, the study was focused on positioning and immediate postprocedural issues. We did not include a follow-up to identify delayed complications (thrombosis or CLABSI). Second, we lacked information about the implications of the extracorporeal part of the catheter for post-procedure nursing care. Third, the PICC-length estimation was based on a model that assumed that the central vascular length would be the same on the right and left sides. However, our results showed a clear discrepancy in catheter lengths between the two sides. This finding highlighted the need for future studies to investigate a more robust solution for venous pathway appraisals.

## 5. Conclusions

In conclusion, our results showed that ECG-guidance technologies were significantly different regarding catheter tip placement; however, this difference had minimal clinical impact. Nevertheless, we found important drawbacks with each technique at the PICC insertion point: the extracorporeal catheter section was longer with WT, and hemostasis was delayed with ST.

## Figures and Tables

**Table 1:** Baseline demographic and clinical characteristics of patients that underwent PICC ^1^ placement with the saline or wire guidance technique.

Characteristic	Saline (*n* = 160)	Wire (*n* = 159)	*p* Value
Demographics			
Men	96 (60)	98 (61.6)	0.819
Age (years)	62.08 ± 15.3 {25–97}	61.01 ± 16.0 {18–93}	0.768
Anatomical considerations			
Left arm access	113 (70.6)	109 (68.6)	0.716
Basilic vein access	107 (66.9)	104 (65.4)	0.814
Therapeutic indications			
Antibiotic therapy	115 (71.9)	155 (72.3)	
Repeated blood draws/Low venous capital	14 (8.8)	8 (5.0)	
Chemotherapy	12 (7.5)	21 (13.2)	
Parenteral nutrition	13 (8.1)	7 (4.4)	
Other	6 (3.8)	8 (5.0)	0.189

^1^ PICC: peripherally inserted central catheter. Values are the number (%) or the mean ± SD {range}, unless otherwise indicated.

**Table 2:** Overall procedure parameters for PICC ^1^ placement with saline or wire guidance.

Procedural Assessment	Saline	Wire		*p* Value
Analyzed patients	160	159		
Technical Success	160 (100)	159 (99.4)		1.000
J wire assistance	12 (7.5)	4 (2.5)		0.070
Analyzed patients	160	159		
Procedure duration (min)	40.3 ± 8.0 {23–67}	36.1 ± 7.2 {18–54}		<0.001
Analyzed patients	147	145		
Intraprocedural recut	0 (0)	80 (55.2) {0–11}		<0.001
Placement side		Wire left side	Wire right side	
Analyzed patients	N/A	99	46	
Intraprocedural recut	N/A	48 (48.5)	32 (69.6)	0.018
Analyzed patients	147	130		
Wave ratio (P wave/QRS complex)	87.1 ± 40.3 {13.3–247.3}	81.5 ± 37.3 {18.8–224.1}		0.124
Combined	84.45 ± 38.9			
P5 *	39.05			
P10 *	46.16			
P25 *	59.54			
P50 *	78.13			

^1^ PICC: peripherally inserted central catheter. Values are the number (%) or the mean ± SD {range}, unless otherwise indicated. P *: indicates the percent of the distribution with a wave ratio below or equal to P wave/QRS complex value.

**Table 3:** Positioning assessments for PICC ^1^ placements with saline or wire guidance.

Procedure Assessment	Saline	Wire	*p* Value
Analyzed patients	160	159	
Navigation and localization success			
T1	58 (36.3)	76 (47.8)	
T2	89 (55.6)	69 (43.4)	
T3	13 (8.1)	14 (8.8)	0.083
Analyzed patients	160	159	
Supplementary actions			
None	147 (91.9)	145 (91.2)	
Repositioning	3 (1.9)	6 (3.8)	
Recut	10 (6.2)	8 (5.0)	0.522
Analyzed patients (T1-T2 classes)	147	145	
DCAJ distance			
Absolute distance (cm)	1.36 ± 0.86	1.12 ± 0.80	0.012
95% CI	1.22–1.50	0.98–1.25	
range	0 to 3	0 to 2.9	
Analyzed patients (T1-T2 classes)	143	142	
Pull-out distance			
Distance (mm)	17.5 ± 9.51	15.81 ± 7.55	0.207
95% CI	0.107–0.386	−0.08–0.181	
range	5–50	5–40	
Combined	16.6 ± 8.6		

^1^ PICC: peripherally inserted central catheter. Values are the number (%) or the mean ± SD unless otherwise indicated.

**Table 4:** Insertion-point assessments of PICC ^1^ placed with saline or wire guidance.

Assessment	Saline (*n* = 147)	Wire (*n* = 145)	*p* Value
Hemostasis			
Direct	74 (50.3)	120 (82.8)	
Delayed	73 (49.7)	25 (17.2)	<0.001
Extracorporeal catheter section, before recut (first insertion)			
E1	147 (100)	40 (27.6)	
E2	0 (0)	56 (38.6)	
E3	0 (0)	49 (33.8)	<0.001
Absolute length (cm)	0.01 ± 0.116	4.36 ± 2.45	<0.001
95% CI	−0.005–0.03	3.96–4.76	
range	0–1	0–11	
Extracorporeal catheter section, after recut			
E1	147 (100)	116 (80)	
E2	0 (0)	29 (20)	<0.001
Absolute length (cm)	0.01 ± 0.12	1.61 ± 1.00	<0.001
95% CI	−0.005–0.03	1.44–1.77	
range	0–1	0–4	

^1^ PICC: peripherally inserted central catheter. Values are the number (%) or the mean ± SD, unless otherwise indicated.

## Data Availability

Data are contained within the article.
